# Arsenic Contaminated Groundwater and Its Treatment Options in Bangladesh

**DOI:** 10.3390/ijerph10010018

**Published:** 2012-12-20

**Authors:** Jia-Qian Jiang, S. M. Ashekuzzaman, Anlun Jiang, S. M. Sharifuzzaman, Sayedur Rahman Chowdhury

**Affiliations:** 1 School of Engineering and Built Environment, Glasgow Caledonian University, Glasgow, Scotland G4 0BA, UK; E-Mails: s.m.ashekuzzaman@gcu.ac.uk (S.M.A.); anlunj@yahoo.co.uk (A.J.); 2 Institute of Marine Sciences and Fisheries, University of Chittagong, Chittagong 4331, Bangladesh; E-Mails: sharifuzaman@yahoo.com (S.M.S.); sayedurrchowdhury@gmail.com (S.R.C.)

**Keywords:** arsenic contamination and removal, developing country, ground water, public health

## Abstract

Arsenic (As) causes health concerns due to its significant toxicity and worldwide presence in drinking water and groundwater. The major sources of As pollution may be natural process such as dissolution of As-containing minerals and anthropogenic activities such as percolation of water from mines, *etc*. The maximum contaminant level for total As in potable water has been established as 10 µg/L. Among the countries facing As contamination problems, Bangladesh is the most affected. Up to 77 million people in Bangladesh have been exposed to toxic levels of arsenic from drinking water. Therefore, it has become an urgent need to provide As-free drinking water in rural households throughout Bangladesh. This paper provides a comprehensive overview on the recent data on arsenic contamination status, its sources and reasons of mobilization and the exposure pathways in Bangladesh. Very little literature has focused on the removal of As from groundwaters in developing countries and thus this paper aims to review the As removal technologies and be a useful resource for researchers or policy makers to help identify and investigate useful treatment options. While a number of technological developments in arsenic removal have taken place, we must consider variations in sources and quality characteristics of As polluted water and differences in the socio-economic and literacy conditions of people, and then aim at improving effectiveness in arsenic removal, reducing the cost of the system, making the technology user friendly, overcoming maintenance problems and resolving sludge management issues.

## 1. Introduction

Due to its significant toxicity arsenic (As) is a global concern as a pollutant of drinking water and groundwater, which has been reported in the USA, China, Chile, Bangladesh, Nepal, Vietnam, Taiwan, Mexico, Argentina, Poland, Italy, Finland, Spain, Canada, Hungary, New Zealand, Japan and India [1,2]. The major sources of As pollution may be natural process such as dissolution of As containing bedrock/minerals and anthropogenic activities *e.g.,* percolation of water from mines, wood preservatives, agricultural chemicals and discharge from uncontrolled mining and metallurgical industry [3,4]. Due to its carcinogenic effects (e.g., cancers of skin, liver, lung, bladder), cardiovascular and neurological effects, and the immune problems of As after long-term or high-dose drinking water exposure, the European Union (EU), the United States (US) and the World Health Organization (WHO) have established a value of 10 µg As/L as the maximum contaminant level for total As in potable water [5,6,7].

Bangladesh is one of countries most affected by As contamination. More than 60% of the groundwater in Bangladesh contains naturally occurring As, with concentration levels often significantly exceeding 10 µg/L [6,8]. In recent decades, some 35–77 million people in Bangladesh have been exposed to toxic levels of arsenic from drinking water [9], while the total estimated population is about 164 million [10]. The sole dependency on groundwater for drinking purposes in rural Bangladesh, which represents nearly 75% of total population, has made the situation much worse.

From the 1970s, in order to avoid using pathogen-contaminated surface water and to reduce waterborne diseases, the Bangladesh government, with the assistance from international aid agencies, began installing shallow (<150 m deep) groundwater tubewells throughout the country [11,12]. Since then, the number of hand pump tubewells has increased exponentially, reaching about 10 million in total [13]. Consequently, 97% of the population in Bangladesh depends on groundwater to access safe water via shallow tubewell extraction systems, which are favoured for the easy availability of aquifers, low-technology installation procedure and affordable cost [14]. Moreover, 85% of the total extracted groundwater is used for irrigation purposes to grow rice and vegetables in the dry season. Unfortunately, much of the groundwater extracted from these shallow alluvial aquifers is often enriched in arsenic. After an extensive As contamination episode in West Bengal, India during 1983, the Department of Public Health Engineering (DPHE) first detected As in well water in the western part of Bangladesh in 1993 [15,16], but the real scale of As contamination in Bangladesh was essentially unknown until 1998 when it was revealed during an International Conference on Arsenic held in Dhaka that 66% and 51% of the 8,065 tubewell water samples collected from 60 out of 64 districts contained As levels above the Bangladesh (50 µg As/L) and WHO (10 µg/L As) standards for drinking water, respectively [17]. In 1999, the British Geological Survey (BGS) along with DPHE reported that 46% and 27% of the 3,534 analyzed tubewells (which amounts to about 0.03–0.05% of all tubewells) across the country, excluding the Chittagong Hill Tracts, exceeded the As guideline value, and an estimated 57 and 37 million people are at risk of exposure to unacceptable drinking water As levels of above 10 µg/L and 50 µg/L, respectively [18]. Recently, Chakraborti *et al*. [19] have presented a complete picture of groundwater As poisoning of all 64 districts in Bangladesh. Based on the water analysis of 52,202 hand tubewells (about 95% samples were collected and processed from 1996–2002), the authors noted that 42.1% and 27.2% of the samples had As concentrations above 10 µg/L and 50 µg/L, respectively, and 7.5% of well waters surprisingly contained As above 300 µg/L, the threshold concentration associated with arsenical skin lesions [20]. Moreover their study mentioned that, approximately 80 million people were vulnerable to groundwater As contamination above the WHO permissible limit of 10 µg/L.

Therefore, it is an urgent need to provide Bangladesh rural households with As-free drinking water. In this regard, different approaches could be applied such as treatment of surface water (typically low in As), rainwater harvesting, treatment of pond waters, use of deeper (>150 m deep) wells to extract low-As groundwater, and above all, exploring low-technology, low-cost, locally fitted systems for arsenic removal from groundwater, but all of these approaches are associated with some practical problems in terms of applicability, economy, infrastructure requirements, generic dissemination and future sustainability, although the last one has received considerable attention over the last two decades due to the possibility of wide-scale application in the field. For example, treatment of surface water involves setting-up industrial-scale water purification and distribution plants which are expensive, time-consuming and investment-intensive, particularly for rural Bangladesh [13,21]. Since water extracted from deeper aquifers typically contains much lower As concentrations [22], exploitation of such aquifers in the Bengal Basin could serve as a source of As-safe water for domestic use in the short-term or on a limited basis [23,24]. This is because most of the Bengal Basin is highly vulnerable to downward migration of high-As shallow groundwater caused by increased withdrawals of deeper groundwater for irrigation [25]. Rainwater harvesting can be used as a source of clean water for use by individual households, but the initial cost to install such a system may not be feasible for many rural families [13,26]. Moreover, it may not be a feasible option to abandon millions of As-contaminated shallow tubewells, rather than finding a suitable As-remediation technology to utilize these existing wells as clean water sources.

Several arsenic removal technologies have been tested so far for As remediation, including ion exchange, activated alumina, reverse osmosis, membrane filtration, modified coagulation/filtration, and enhanced lime softening [27,28]. However, for any effective technology to be appropriate for use in affected areas of developing countries like Bangladesh, it should ideally be simple, low cost, versatile, transferrable, and should be adaptable to both point of use (PoU) household units and community-based application [26]. In this regard, Berg *et al*. [28] mentioned that none of the US Environmental Protection Agency (USEPA) recommended technologies are practical in low income regions as they require sophisticated technical systems and hence, these are not currently applied on a broad scale in developing countries. The most common and useful technologies that have been utilized for As removal in Bangladesh as well as in other developing countries are based on oxidation, co-precipitation and adsorption onto coagulated flocs, and adsorption onto sorptive media [29]. According to Jiang [30], these technologies could be the cost-effective for removing As for the developing world. 

Considering the serious arsenic contamination in Bangladesh groundwater and the fact there is scarce literature providing an overview on the recent arsenic contamination status and feasible treatment technologies, this paper then aims to review comprehensively the recent arsenic As contamination status, its sources and reasons for mobilization, exposure pathways in Bangladesh as well as As removal technologies that are to be applied in Bangladesh, making this a potentially useful resource for researchers or policy makers to identify and investigate the most promising treatment options.

## 2. Arsenic in Water: Occurrence and Speciation

As is found in aqueous environments predominantly in the 3^+^ (As(III), arsenite) and 5^+^ (As(V), arsenate) oxidation states, however, other oxidation states *i.e.*, 0 (arsenic), 3^−^ (arsine) can also be exist. It mainly occurs due to natural and anthropogenic reasons. Among the natural sources, arsenic disulphide or realgar (As_2_S_2_), arsenic trisulphide or orpiment (As_2_S_3_) and arsenopyrite or ferrous arsenic sulphide (FeAsS) are considered as the main mineral sources of As, although there are over 245 species of As-containing minerals [12]. As can be determined in water both in the inorganic and organic forms, while the latter are quantitatively insignificant and are found mostly in either waters or in areas severely affected by industrial pollution [31]. Inorganic arsenic species (arsenite and arsenate) are the dominant forms that are usually detected in groundwater and these are more toxic than organic ones (e.g., methylated arsenic) [15].

The speciation of inorganic As species is important when considering toxicological studies and remediation techniques, particularly since the latter is highly species dependent. Two factors (pH and redox potential) are very important in controlling As speciation [2]. As^5+ ^species (H_3_AsO_4_, H_2_AsO_4_^−^, HAsO_4_^2^^−^, and AsO_4_^3^^−^) are dominant under oxidising conditions, with H_2_AsO_4_^−^ dominates at low pH (pH < 6.9) and HAsO_4_^2^^−^ at higher pH, while H_3_AsO_4_ and AsO_4_^3^^−^ may occur in extremely acidic and alkaline conditions, respectively. On the contrary, As^3+^ species (H_3_AsO_3_, H_2_AsO_3_^−^, and HAsO_3_^2^^−^) are stable under reducing (or anoxic) conditions, with the most common being the uncharged species H_3_AsO_3_ at pH < 9.2 [2,31].

The ratio of As^3+ ^to As^5+ ^in groundwaters is highly unstable due to the variations in the availability of redox active solids, especially organic carbon, the activity of microorganisms, and the extent of convection and diffusion of oxygen from the atmosphere. For example, the proportion of As^3+ ^in As-rich reducing groundwater of Bangladesh is about 10–90% of the total As concentration, with typical ranges around 50–60% [31]. Due to the presence of uncharged As^3+ ^species in reducing environments under a wide range of pH values (0–9), most of the sorption techniques can hardly remove As^3+^. In this regard, the contaminated water where As^3+ ^is the dominant species needs to be pre-treated by oxidation. In the presence of sulphide, dissolved As-sulphide species can be significant due to precipitation of orpiment (As_2_S_3_), realgar (AsS) or other sulphide minerals containing coprecipitated As under favourable reducing and acidic conditions, and thereby, considerable control over trace As-concentration can be achieved [30,31]. Moreover, As reacts with iron and manganese hydroxides, when these oxides are present in both groundwater and surface water, and subsequently is immobilized through adsorption-coprecipitation. However, As is susceptible to mobilize when As coprecipitated iron or manganese solids are dissolved under reducing conditions, or released from the precipitate surfaces in the presence of orthophosphate and natural organic matter (NOM) due to competition for sorptive surface sites [30].

## 3. Contamination Level of As and Vulnerable Areas

**Figure 1:**
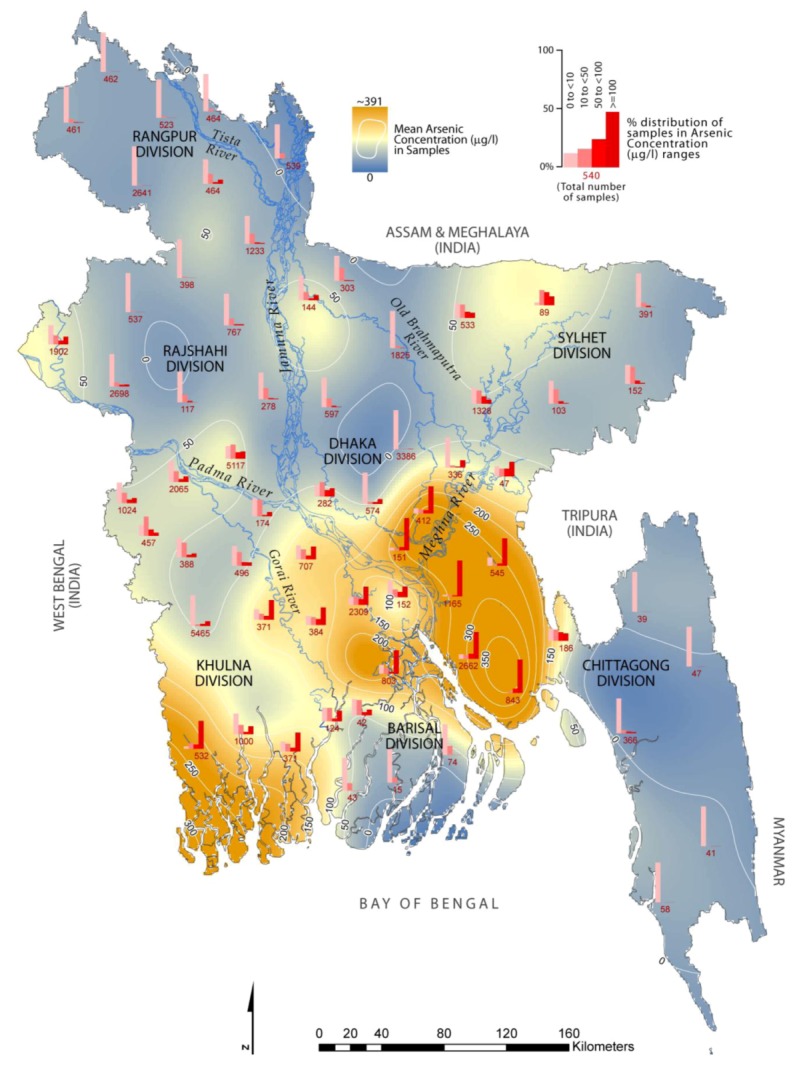
Arsenic (As) concentrations in groundwater of Bangladesh, showing all 64 districts under seven administrative divisions. N = 52,202 tubewells samples; where n = 6,787, 11,814, 12,169, 1,101, 5,999, 13,597 and 735 were representative samples of Rangpur, Rajshahi, Khulna, Barisal, Chittagong, Dhaka and Sylhet divisions, respectively (data source: [19]).

The distribution of As with mean concentration (as shown by contour lines) in groundwater of Bangladesh is presented in Figure 1, where the bar diagrams beside each of the 64 districts represent the contamination levels by showing percentage distribution of analyzed tubewells samples in four As concentration ranges (<10 to ≥100 µg/L). It is seen from Figure 1 that almost all of the surveyed districts are contaminated with As (>10 µg/L), except for four districts (Rangamati, Khagrachari, Bandarban and Cox’s Bazar) out of 64 from the Hill tract regions of Chittagong division. However, ten contaminated districts out of 60 under the Rangpur (Panchagarh, Kurigram, Lalmanirhat, Nilphamari, Dinajpur), Rajshahi (Joypurhat, Naogaon) and Barisal (Barguna, Bhola, Patuakhali) divisions, respectively, showed that a limited percentage (1–20%) of analyzed samples were contaminated in the range of 10–50 µg/L. The most affected regions with As contaminated aquifers are the southeast (Meghna floodplains) and southern (coastal area of Khulna division) parts of Bangladesh (Figure 1). The major affected districts are distinguished based on the frequency (>50%) of the samples identified with As concentrations of more than 50 µg/L and among these, Satkhira, Munshiganj, Chandpur, Laksmipur, Noakhali can be regarded as the most vulnerable to As contamination due to their abundant availability of groundwater samples with As concentrations ≥100 µg/L (observed from 70–90% of the total analyzed samples) (Table 1). These findings based on the data from [19] are in good correspondence with others [12,15,18], thus confirming the updated As contamination status in Bangladesh. In this respect, one important matter to consider is the method of As detection in water samples, because both field-kit and laboratory tests were employed by many government and non-government organizations to identify the As contaminated water wells. Although both of the tests provide the same indication of the general geographical distributions of As contamination, field test kits have the limitation of detecting As contaminated groundwater with concentrations between 50 and 200 µg /L [32], so the current study is perhaps giving a reliable picture of the As contamination scenario (Figure 1), since it involves test results based on laboratory analysis of 52,202 representative water samples from all 64 districts of Bangladesh. According to Chakraborti *et al*. [19], the estimated population exposed to As contaminated water above 10 and 50 µg/L, could be above 36.6 million from 59 districts and 22.7 million from 50 districts, respectively. Ironically, a real fact is that one in five deaths in Bangladesh could be attributed to As exposure (>10 µg/L) in drinking water, as published recently in *The Lancet* from a 10 year study conducted by international team of researchers, and the WHO has described the phenomenon of As crisis in Bangladesh as “the largest mass poisoning of a population in history” [9].

**Table 1:** Major As contaminated districts with >50% analyzed samples exceeding Bangladesh guideline value (50 µg/L) [19].

Division	District (number of sampled wells in parenthesis)	Severely affected with As concentration ≥100 µg/L (% samples)
Khulna	Bagherhat (371), Narail (371), Satkhira (532)	Satkhira (~70%)
Barishal	Barisal (803)	Barisal (~59%)
Sylhet	Sunamganj (89)	*
Dhaka	Gopalganj (384), Madaripur (2,309), Munshiganj (151), Narayanganj (412)	Munshiganj (~80%)
		Narayanganj (~68%)
Chittagong	Brahmanbaria (47), Chandpur (1,165), Comilla (545), Lakshmipur (2,662), Noakhali (843)	Chandpur (~90%)
		Lakshmipur (~70%)
		Noakhali (~85%)
		Comilla (~69%)

* The percentage of analyzed samples in the given region was less than 50% for exceeding Bangladesh guideline value (50 µg/L).

## 4. Sources, Distribution and Mobility of As

It is now accepted that the major sources of arsenic contamination in the groundwater of Bangladesh are geological deposits, and its release occurs through natural processes [12,32,33], although a number of anthropogenic sources (e.g., use of fertilizers, pesticides, insecticides, and herbicides containing arsenic; use of arsenic compounds as preservatives in wooden electric poles of the Rural Electrification Board; tubewell filters coated with arsenic compound; industrial waste disposal; and enhanced leaching beneath irrigated lands) were initially considered for such occurrence [15]. The As-enriched aquifers are generally shallow (<100 m deep), of Holocene age and consist of unconsolidated grey micaceous sands, silts and clays deposited as alluvial and deltaic sediments by the Ganges, Brahmaputra and Meghna (G-B-M) rivers [23,31]. The G-B-M river system originated from the Himalayas and acts as an important drainage system in the Bengal Delta Plain (BDP) by channelling suspended solids (1,060 million tons), water (1,330 km^3^) and dissolved particulates (173 million tons) to the Bay of Bengal [34]. Thus, the Bay of Bengal receives a maximum amount of sediments from this river system containing several trace elements, including arsenic.

Although it is largely agreed by the researchers that dissolved arsenic in the groundwater of Bangladesh originates from the sediments, there is no evidence of widespread, unusually high levels of solid phase arsenic in the aquifer material [35], except the range reported as <2–20 mg/Kg; only slightly higher than typical sediments (2–6 mg/kg) [32]. So, this leads to the fact that high dissolved arsenic in groundwater is associated with particular hydrologic and biogeochemical conditions that partition arsenic from the solid to aqueous phase, with the existence of arsenic in a potentially soluble form. The distribution of arsenic in Bangladesh groundwater is closely related to the major geo-morphological units, namely: Tertiary hills, Pleistocene uplands and Holocene plains, and the As-enrichment is mainly restricted to the Holocene alluvial aquifers at shallow and intermediate depths [36]. This corresponds well with the most As-affected regions (Figure 1) in Bangladesh, *i.e.*, central, south and south-eastern parts that are essentially Holocene deltaic and flood plains under the Padma-Meghna sub-basin. The lithology of these areas is composed of grey clay, silt, fine sand with occasional peats and gravels. The aquifers are generally characterized as highly transmissive and multi-layered, varying from unconfined to leaky-confined in the shallow alluvial deposits, and confined in the deeper alluvial deposits [37]. The strata of the aquifer system can be differentiated as (from top to bottom): upper aquifer (shallow) or composite aquifer composed of very fine to fine sand (extends down to 40–60 m below ground surface); main aquifer, known as main water-bearing zone, and consisting of medium to coarse sand with occasional gravel (occurs down to a depth of about 140 m), and a deeper aquifer or deeper water-bearing unit that occurs at depths >150 m, and consists of medium to coarse sand interbedded with fine sand, silt and clay. This zone is separated from the overlaying main aquifer by one or more clay layers of variable thickness [38]. However, much of the aquifer system in Bangladesh is simply known as shallow aquifer (typically extending from 10 to 70 m below ground level) and deeper aquifer below about 200 m [32]. Groundwater samples from depths between 20 and 50 m were observed to have the maximum As concentrations, whereas those from depths shallower than 10 m and deeper than 150 m tend to be mainly As-free [18]. Such variation and availability of As concentrations with depth can be explained by resorption of As onto residual iron-oxyhydroxides (FeOOH) [39], and by the nature of the aquifer sediments [40]. Moreover, it is reported that mineralogical characteristics of the sediments and their interactions with the aqueous phase under varying redox states reflect the differential concentrations of As [34].

In Bangladesh, the mode of occurrence and distribution of As in groundwater aquifers has been studied widely in the recent decades. In this regard, various geo-chemical parameters of the subsurface aquifers and their relationships with As have been determined in a number of studies to understand the mobility mechanism of As in groundwater [23,40,41,42,43,44]. Although several hypotheses, including: (1) reductive dissolution of iron-oxyhydroxides which releases sorbed As; (2) oxidative dissolution of As-rich pyrite, and (3), anion exchange of sorbed As by increasing concentration of phosphate (PO_4_^3^^−^) from fertilizers, have been proposed to explain the problem of As-enrichment, the right one is still not clear [45]. However, it has been widely reported that microbially mediated reductive dissolution of Fe(III) oxyhydroxides (*i.e.*, hypothesis 1), facilitated by electron donors, e.g., organic matter and/or dissolved organic carbon (DOC), under moderate to strong reducing conditions is the primary mechanism to release As in groundwater of the Holocene deltaic aquifers [36,37,41,42]. At the beginning of the As problem in Bangladesh, it was an accepted hypothesis that oxidation of arsenic-rich pyrite by atmospheric oxygen act was the dominant process to mobilize As due to lowering of water table following excessive pumping of groundwater [36], but the reducing environment of subsurface aquifers with low concentrations of SO_4_^2^^−^ in groundwater samples, and the inverse relationship between dissolved As and SO_4_^2^^−^ concentrations, suggest that pyrite/sulfide oxidation is not the major process for As release [43,44]. Moreover, if pyrite oxidation were true, it’s As would be occluded in the resulting Fe-oxyhydroxide, rather than released to groundwater [46]. Nevertheless, some other factors, such as stimulated microbial activity by inflow of organic carbon from excessive withdrawal of groundwater due to irrigation pumping, competition for sorption sites with As by relatively high concentrations of phosphate from application of phosphate fertilizer, and dissolution of other solid phases (e.g., phyllosillicate minerals) can also be associated with As mobilization in Bangladesh groundwater [36,42].

**Table 2:** Average groundwater composition of the most As contaminated areas* in the Bengal Delta Plain (BDP) in Bangladesh.

Aqueous parameters	Measured range^+^ (*Mean value ± SD*)^++^	
	Shallow aquifer (10–69 m), N = 89	Deep aquifer (70–260 m), N = 34
pH	6.4–7.9 (*7.1 ± 0.2*), n = 88	6.5–7.3 (*7.0 ± 0.2*), n = 34
EC (µS/cm)	410–3,650 (*1,003.7 ± 637.7*), n = 60	317–3,410 (*960.8 ± 638.7*), n = 28
ORP (mV)	+95 to −2 (*30.1 ± 28.4*), n = 27	24–90 (*46.2 ± 22.0*), n = 9
DO (mg/L)	<0.1–2.1 (*0.6 ± 0.8*), n = 39	<0.1, n = 9
Na^+^ (mg/L)	8–480 (*48.4 ± 71.5*), n = 72	7.9–280 (*62.3 ± 61.2*), n = 28
K^+^ (mg/L)	2.4–20 (*7.2 ± 3.4*), n = 72	3.2–26.1 (*7.7 ± 5.2*), n = 28
NH_4_^+^ (mg/L)	0.7–19.9 (*7.9 ± 5*), n = 56	0.1–10.3 (*2.8 ± 2.9*), n = 15
Ca^2+^ (mg/L)	12–174.1 (*82.4 ± 41.5*), n = 83	7–211 (*59.2 ± 41.3*), n = 32
Mg^2+^ (mg/L)	11–105.7 (*29.1 ± 17.0*), n = 72	14–110 (*30.6 ± 19.7*), n = 28
HCO_3_^−^ (mg/L)	220–931.4 (*494.5 ± 134.2*), n = 72	184–697 (*359.7 ± 137.4*), n = 28
Cl^−^ (mg/L)	1.9–695 (*46.3 ± 91.3*), n = 72	1.5–797 (*97.1 ± 161.8*), n = 28
NO_3_^−^ (mg/L)	<0.03–5.9 (*1.2 ± 1.7*), n = 72	<0.03–7.1 (*2.0 ± 2.3*), n = 29
SO_4_^2^^−^ (mg/L)	<0.01–34 (*3.4 ± 6.4*), n = 72	<0.01–46 (*8.1 ± 10.8*), n = 29
PO_4_^3^^−^ (mg/L)	0.46–15 (*4.9 ± 3.2*), n = 60	0.05–5.5 (*1.5 ± 1.6*), n = 29
As (µg/L)	22–1,000 (*373.7 ± 244.1*), n = 89	0.2–170 (*48.0 ± 52.6*), n = 34
Fe (mg/L)	0.06–22.2 (*7.2 ± 4.8*), n = 89	0.01–17.5 (*3.0 ± 4.2*), n = 34
Mn (mg/L)	0.02–2 (*0.7 ± 0.5*), n = 43	0.06–2.9 (*0.6 ± 0.8*), n = 22
DOC (mg/L)	0.64–15 (*4.3 ± 3.0*), n = 72	0.2–12 (*2.5 ± 2.6*), n = 28

EC = electrical conductivity, ORP = oxidation reduction potential, DO = dissolved O_2_, DOC = dissolved organic carbon, SD = standard deviation; * The sample locations include the As-affected areas under the districts of Noakhali (n = 2), Magura (n = 2), Brahmanbaria (n = 7), Laksmipur (n = 10), Munshiganj (n = 31), Faridpur (n = 11), Chandpur (n = 23), Narayanganj (n = 35) and Jhenaida (n = 2) along the eastern margin of the Bengal Basin (Padma-Meghna sub-basin, N = 123); ^+^ Data source: [16,37,41,43,44]; ^++^ Values calculated based on the mentioned data source.

An average groundwater composition for the locations recognized as mostly As contaminated along the eastern margin of the Bengal Basin under the Padma-Meghna sub-basin is given in Table 2. Under the shallow aquifer (N = 89), 93% of the groundwater sampling points had As concentrations of more than 50 µg/L (the allowable limit of As in Bangladesh), while the same figure was 44% in the case of deep aquifers (N = 34). In the deep aquifers, most of the As contamination occurred between the depths of 70 to 100 m, while depths >150 m were mainly As-free, so it is believed that Table 2 provides a reliable picture of the groundwater composition of the As-enriched aquifer, having a good number of representative data from [37,41,43]. From Table 2, it is revealed that the groundwater is characterized by *circum*-neutral pH with a moderate to strong reducing nature, as demonstrated by low concentrations of nitrate (NO_3_^−^) and sulfate (SO_4_^2^^−^), and high concentrations of DOC, dissolved iron (Fe^2+^) and ammonium (NH_4_^+^) ions (Table 2). Moreover, the ORP value ranged between +95 to −2 mV, clearly indicating the reduced conditions, as the value between 0‒200 mV is categorized as reduced condition [47,48]. The waters are generally of Ca-Mg-HCO_3_ or Ca-Na-HCO_3_ type, with HCO_3_^−^ as the dominant anion (220–931 mg/L). The distribution of the major cations such as Ca (12–174 mg/L), Mg (11–106 mg/L), Na (8–480 mg/L) and K (2.4–20 mg/L) shows significant variations with depth as well as by region. Considerable differences are also observed in the concentration range of As (22–1,000 µg/L), Fe (0.06–22 mg/L) and Mn (0.02–2 mg/L) as a function of both depth and region in the groundwater samples (Table 2), which can be attributed to varying redox states at shallow depth within the aquifer. The concentration of PO_4_^3^^−^ is observed to be in the range between 0.46 to 15 mg/L (Table 2), and this may result either from microbially mediated reductive dissolution of Fe(III)-oxyhydroxide [39] or from widespread application of P enriched fertilizers [34,43]. The correlation between As and other potential aqueous parameters has been well documented, and it was found that there exists a moderate to strong positive correlation between the pairs of As and Fe (r^2^ = 0.6), As and HCO_3_^−^ (r^2^ = 0.71), As and PO_4_^3^^−^ (r^2^ = 0.75), As and DOC (r^2^ = 0.83), Fe and PO_4_^3^^−^ (r^2^ = 0.67), Fe and DOC (r^2^ = 0.72), DOC and PO_4_^3^^− ^(r^2^ = 0.83), and DOC and HCO_3_^−^ (r^2^ = 0.57), respectively [43]. Moreover, low concentrations of both NO_3_^−^ and SO_4_^2^^−^ with negative correlation with As (r^2 ^= −0.36 and r^2^ = −0.096, respectively) were observed [44]. These relationships demonstrate that reductive dissolution of Fe(III)-oxyhydroxides with microbially mediated degradation of organic matter acts as the dominant mechanism of As release to groundwater.

## 5. Exposure Pathways and Health Effects of Arsenic

**Figure 2:**
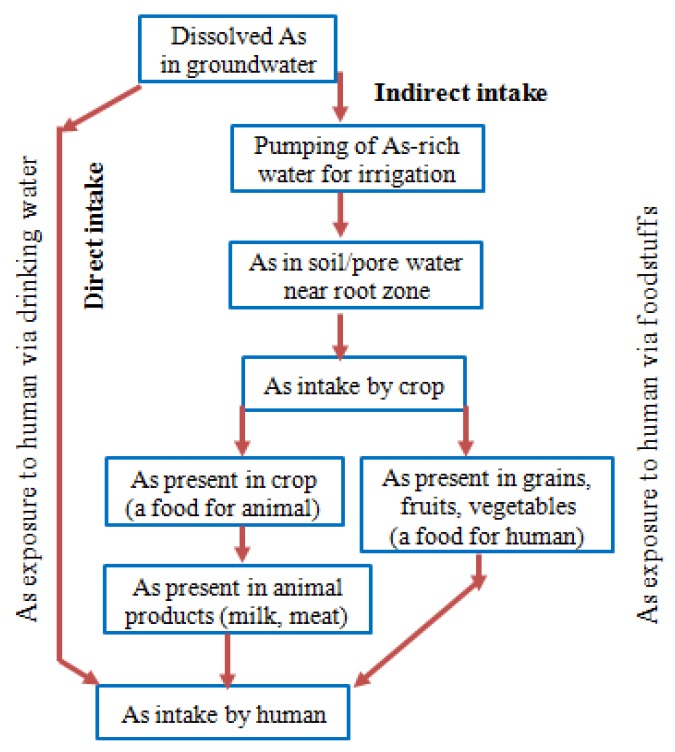
Flow chart of arsenic (As) intake by human (after [51]).

Arsenic (As) is a Class “A” human carcinogen as classified by the USEPA and its presence in the environment at specific concentrations, either as a dissolved contaminant in water, food web bioaccumulation, or as an inhaled particulates in the atmosphere, is of great concern to the wellbeing and health security of humans, with the situation being at its worst in Asia [49,50]. The ingestion of As into the human body takes place either by direct intake of arsenic rich water (*via* drinking or cooking foods) or by indirect intake through crops grown using arsenic-contaminated water. A flow chart of such arsenic exposure pathways is shown in Figure 2.

For example, through direct drinking of arsenic contaminated water with a concentration at 50 µg/L, an adult would become intoxicated with 200–300 μg of As per day, because the average range of water consumption by the adult of Bangladesh is 4–6 litres in a day [52]. On the other hand, indirect exposure of As through the human food chain is another important issue of concern [53]. In this process As levels in soils can reach up to 83 μg/g in comparison to background levels of As in soils (4–8 µg/g), when the crop lands are irrigated with arsenic-contaminated groundwater [52]. Thus, primarily in the dry season, over 1,000 tonnes of As are transferred to rice fields through irrigation each year in Bangladesh [54]. This factor equally poses a potential public health problem as paddy rice is inherently a more efficient accumulator of As, probably through the highly efficient pathway for silicon, than other cereal crops [49,55]. Rice is the most important cereal and staple food for the people of Bangladesh who eat an average of 450 g rice a day [52], thus ingestion of rice is by far the dominant source (*i.e.*, 50–70% of the total meal) of As poisoning pathway for populations exposed to low or no As in drinking water [50,56]. It is estimated that the daily consumption of rice with a total arsenic level of 0.08 μg/g (dry weight) contributes an equivalent drinking water arsenic level of 10 μg/L [51]. Evidence of As uptake by other foodstuffs has been reported in several studies which also represent potential sources of dietary arsenic exposure [52,53,57]. Of relevance, some vegetables of Bangladesh contained total As levels between 5 and 540 μg/kg, with a mean of 54.5 μg/kg, which was approximately 2- to 3-fold higher than those observed for the vegetables of United Kingdom (U.K.). High amounts of As have also been detected in fishes of freshwater origin (*i.e.*, up to 1,318 μg As/kg, with a mean of 580 μg As/kg), although marine fish and shrimp were shown to have lower levels, *i.e.*, 214–266 μg As/kg [53]. A comparison of As concentrations among different food composites of Bangladesh and other countries are shown in Table 3. In addition, food preparation with As-contaminated water is associated with an increased total As content, thus 10–35% higher arsenic was noted with cooked rice than that of raw rice, in arsenic-contaminated areas of Bangladesh [58]. However, the retention of total As in cooked rice can be kept lower by the use of water containing low level of arsenic, and following the cooking method with “excess water and discarding the concentrated cooking water” [52].

While large-scale arsenic contamination in Bangladesh was first confirmed in 1993, reliable estimates of the magnitude of arsenic exposure and its related health problems are still insufficient [66]. However, epidemiological studies on the public health effects of arsenic exposure from drinking water suggested a carcinogenic effect, evidenced by an increased risk of cancers of the skin, lung, bladder, liver and kidney, and a contaminant level of 50 µg As/L could lead to cancer in 1 in 100 individuals. In the case of chronic poisoning, this deadly element accumulates in hair, skin and nails, resulting in strong pigmentation of hands and feet (*i.e.*, keratosis), high blood pressure, and cardiovascular, respiratory, endocrine, neurological and metabolic dysfunctions/disorders [50,66,67,68,69]. Even exposure to As levels of 8.1–40.0 µg/L has been linked to skin lesions, which often develop within 5–15 years of exposure, although this risk appears to be influenced by host factors such as gender, age and body mass index [70]. Moreover, lower levels of As intake from drinking water, which is a widespread source of exposure worldwide, may play a role in the prevalence of diabetes [71], and significantly impair the immune response to diseases (*i.e.*, respiratory influenza A virus infections in mouse) suggesting an increased risk of lung disease in exposed human populations [72]. An abnormal growth of cells, *i.e.*, neoplastic transformation, may arise due to the chronic ingestion of small amounts of inorganic arsenic (iAs), while a single 100 mg dose of iAs is enough to shut down energy metabolism [73]. If daily consumption of 1.6 liters of water contains iAs concentrations of 50 μg/L then the risk of cancer-death may be 21/1,000 [74]. Due to As exposure during pregnancy (exposure prevalence: 25–50% at drinking water level higher than 50 µg/L), the deaths of infants may reach up to 250,000 per year in Bangladesh [75]. Additional As-induced effects such as fatal loss and adult disease mortality have also been observed [66], with studies pointing out that As can account for 21.4% of all deaths among exposed populations [9]. Nevertheless, the full burden of As exposure in Bangladesh remains to be fully understood.

**Table 3:** Arsenic contents (μg/kg) in food composites from different countries.

Foodstuffs	Total As (μg/kg)	Reference
	**Mean (Range)**	
***Vegetables***		
Bangladesh ^a^	(70–3,990)	[59]
Bangladesh	54.5 (<5–540)	[53]
Europe	(<5–87)	[53]
UK (Food Standards Agency)	2 for green vegetables	[60]
	4.9 for other vegetables	
***Rice***		
Australia	30 (20–40)	[61]
Bangladesh	500 (30–1,840)	[62]
China	140 (20–460)	[57]
West Bengal (India)	140 (20–400)	[63]
USA	250 (30–660)	[57]
Bangladesh ^a^	496 (58–1,830)	[62]
China^a^	930	[64]
West Bengal (India) ^a^	250 (140–480)	[63]
	330 (180–430)	[65]
***Fish and shrimp***		
Bangladesh ^b^	(214–266)	[53]
Bangladesh	(97–1318)	[53]
***Other foods***		
Bangladesh (Betel leaf)	45.9 (44.9–46.9)	[53]

^a^ Samples were collected from arsenic-affected area; ^b ^Marine species.

The assessment of exposure to As typically include measurements of several biomarkers such as blood, hair, nail, or urinary concentrations of arsenic (or metabolites). Blood As is only a useful biomarker in the case of acute arsenic poisoning or steady chronic high-level exposures [69]. Since As is cleared from the bloodstream relatively quickly and excreted via the kidneys, therefore, blood and urine are a good indication of recent exposure to As [76]. Hair and nails levels are suggestive of long-term exposure, but these signs do not provide the information about the dose ingested [50]. The dietary exposure to As from food supply can be known if urine As levels reach 50 mg/L [77]. As a biomarker of early effect, the complementary use of micronuclei assays (or chromosomal aberrations) may provide a better understanding of the toxicity of arsenic-induced health hazards [50].

## 6. Treatment Processes for Remediation of As Contaminated Water

A variety of treatment technologies for the removal of arsenic from ground water have been studied; which were based either on the laboratory or on the site scale work. These technologies are mainly based on oxidation, co-precipitation, sorption, filtration, and ion exchange. The aims of this section are to review and update the recent advances made in the technological development in arsenic removal technologies to explore the potential of those advances to address the problem of arsenic contamination. Especially, attention is paid to address the efficiency and applicability/appropriateness and the economic, robustness and social acceptability of the various technologies.

### 6.1. Co-Precipitation, Coagulation, and Filtration

In the water treatment plants of Hanoi City (Vietnam), activated hypochlorite was introduced after the aeration tank in the conventional water treatment process which was able to oxidise As(III) to As(V) and then to lower arsenic concentrations below the standard level (<0.05 mg/L) with relatively low Fe concentration (5 mg/L) [78]. Pilot scale investigations indicated that the removal efficiency of As in the pilot test system was much higher than that in the laboratory experiments. To reduce As concentrations to the levels lower than the standard level of 0.05 mg/L, initial Fe/As concentration ratios used in the pilot system and laboratory experiment were 16 and 50, respectively.

**Figure 3:**
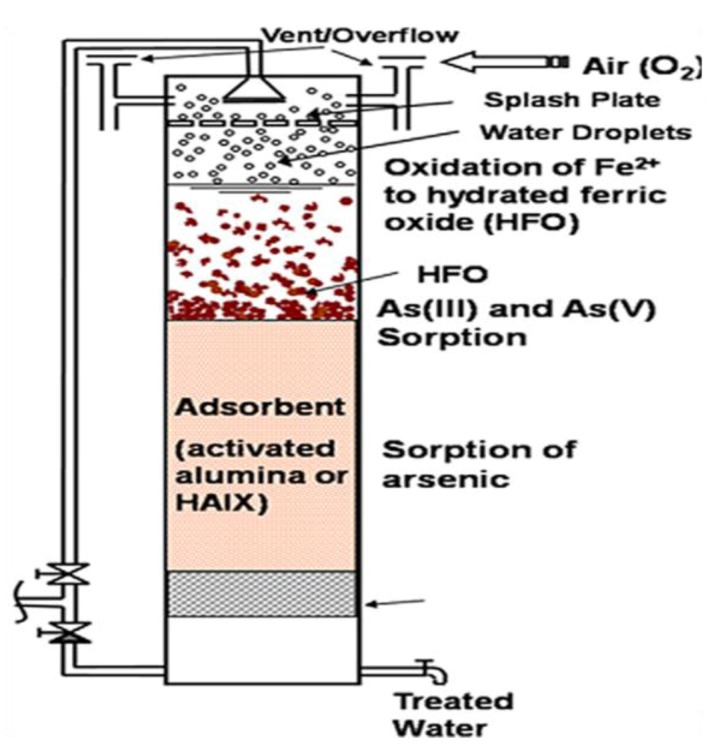
The community-scale arsenic removal unit (after [21]).

Community-scale arsenic removal units were developed and installed in West Bengal (India) to treat arsenic-containing groundwater [21]. Figure 3 shows the unit structure and the associated reactions. It consists of a stainless steel column filled with two types of adsorbents. The raw water inlet is located at the top and the treated water is collected from the bottom of the column. The raw water inlet is designed with a spray head and splash plates so that small water droplets are formed at the entry point. Also, the top part of the column is designed with a large void space with vent connections that allow the air to get dissolved in the water droplets. The dissolved air helps oxidize the ferrous iron in the raw water to form fine precipitates of hydrated ferric oxide (HFO).

Freshly precipitated HFO can selectively bind both arsenites and arsenates through formation of bidentate and/or monodentate inner-sphere complexes where Fe(III) serves as electron-pair acceptor. A significant portion of influent arsenic is adsorbed by the freshly formed HFO precipitates inside the column. The remaining arsenic in the water is removed by a bed of adsorbent provided at the bottom part of the unit. Activated alumina or hybrid anion exchanger (HAIX) [79] were used for most of the units.

The desired treated water flow rate from the arsenic removal unit is 10–12 L/min. The flow rate, however, diminishes over time due to the precipitation of the HFO particles within the bed. Backwashing of the column every other day to drive out the HFO particles is necessary to maintain a good flow rate through the column. The arsenic-laden HFO particles in the waste backwash are trapped on top of a coarse sand filter provided in the same location.

Depending on the arsenic and iron concentration in the raw water, the arsenic removal units produce an average about 10,000 bed volumes of treated water before the concentration of arsenic in the treated water exceeds the maximum contaminant level (MCL) which is currently set at 50 µg/L in India or Bangladesh. Once the arsenic concentration in the treated water exceeds the MCL, the exhausted adsorption media is removed from the unit and taken for regeneration at a nearby central regeneration facility. The operation of the arsenic removal unit is resumed with another adsorption media which is already regenerated. The adsorbents were regenerated in a central facility by a few trained villagers. The process of regeneration reduces the volume of disposable arsenic-laden solids by nearly two orders of magnitude and allows for the reuse of the adsorbent material. Finally, the arsenic-laden solids are contained on well-aerated coarse sand filters with minimum arsenic leaching. The continued safe operation of these units has amply demonstrated that use of re-generable arsenic-selective adsorbents is quite viable in remote locations.

**Figure 4:**
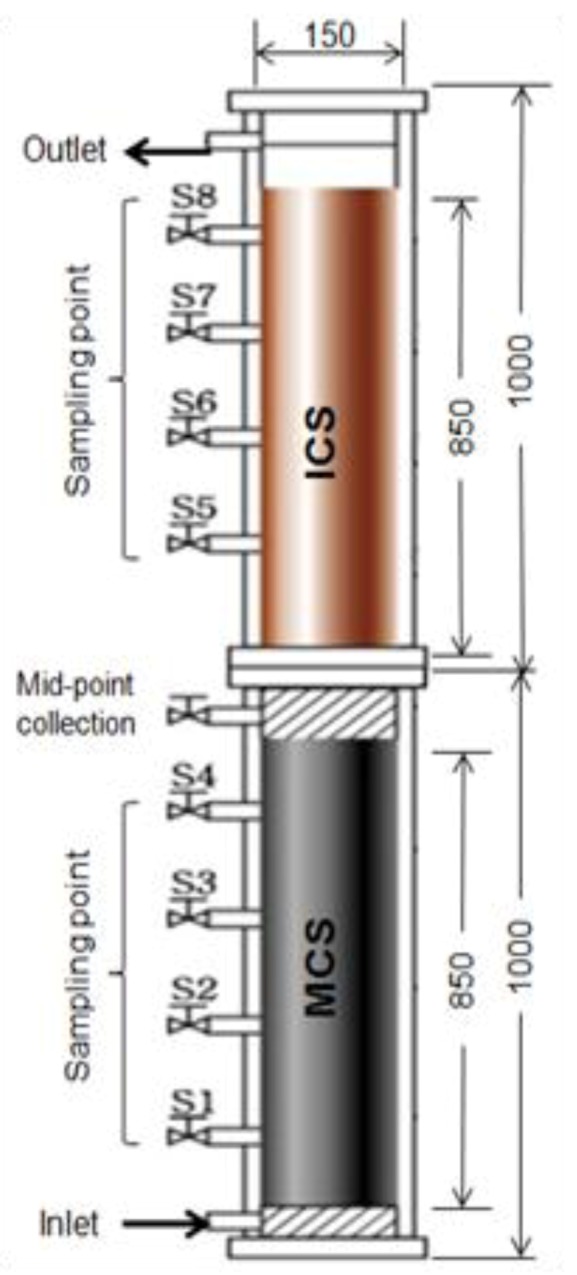
Schematic apparatus of reactive sand filtration tower (RSFT) (scale unit: mm) (initial As(III) = 1.0 mg/L, Q = 22.5 mL/min) (after [80]).

As one of recent development in the field of As removal by filtration, the applicability of manganese-coated sand (MCS) and iron-coated sand (ICS) for the treatment of As(III) via oxidation and adsorption processes was investigated [80]. From a bench-scale column test, a column reactor packed with both MCS and ICS was found to be the best system for the treatment of As(III) due to the promising oxidation efficiency of As(III) to As(V) by MCS and adsorption of As(V) by both MCS and ICS. From these bench-scale results, the treatment of synthetic wastewater contaminated with As(III) was investigated using a pilot-scale filtration system packed with equal amounts (each 21.5 kg) of MCS at the bottom and ICS on the top (Figure 4). The height and diameter of the column were 200 and 15 cm, respectively. As(III) solution was introduced into the bottom of the filtration system, at a speed of 5 × 10^−3^ cm/s, over 148 days. The breakthrough of total arsenic in the mid-sampling (end of the MCS bed) and final-sampling (end of the ICS bed) positions began after 18 and 44 days, respectively, and showed complete breakthrough after 148 days. Although the breakthrough of total arsenic in the mid-sampling position began after 18 days, the concentration of As(III) in the effluent was below 50 μg/L for up to 61 days. This result indicates that MCS has sufficient oxidizing capacity for As(III), and 1 kg of MCS can oxidize 93 mg of As(III) for up to 61 days. When the complete breakthrough of total arsenic occurred, the total arsenic removed by 1 kg of MCS was 79.0 mg, suggesting MCS acts as an adsorbent for As(V), as well as an oxidant for As(III). From this work, a potential full scale filtration system can be used to simultaneously treat As(III) and As(V).

### 6.2. Precipitation and Filtration Unit at Household Scale

**Figure 5:**
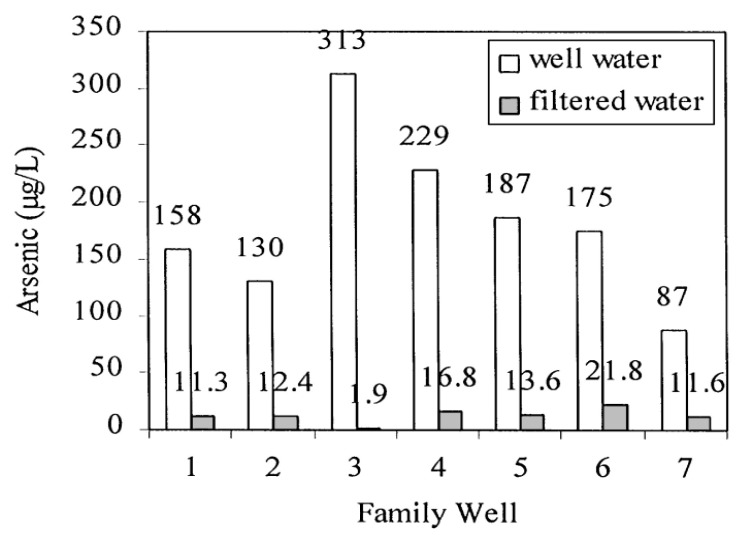
Comparison of arsenic concentrations in well water and the average arsenic concentration in the filtrate (after [81]).

A field study was performed to test a simple household arsenic removal system by precipitation and filtration in seven households in Bangladesh [81]. The household filtration process included co-precipitation of arsenic by adding a packet (approximately 2 g) of ferric and hypochlorite salts to 20 L of well water and subsequent filtration of the water through a bucket sand filter. Ferric hydroxides were required with the Fe/As ratios of greater than 40 (mg/mg) to reduce arsenic to less than 50 μg/L, because the presence of elevated phosphate and silicate concentrations decreased the removal of As due to they compete with As(V) for ferric hydroxide sorption sites. Experimental results obtained from the participating families proved that the household treatment process removed arsenic from approximately 87–313 μg/L in the well water to 1.9–21.8 μg/L in the filtrate, which were much less than the maximum allowable limit of As concentration for Bangladesh drinking water guideline (50 μg/L) (Figure 5).

Following up the demonstration studies described above, a larger scale field test was conducted [13]. A small community arsenic removal system for six households in Bangladesh was used to treat well water containing As (190–750 μg/L), Fe (0.4–20 mg/L) and P (0.2–1.9 mg/L). The system removes As by filtration through a sand bed following the addition of about 1.5 g of ferric sulphate and 0.5 g of calcium hypochlorite. The residual arsenic concentrations in all the treated samples (except one containing 69 μg As/L) were below the Bangladesh drinking water standard of 50 μg/L, and approximately half of the samples also met the World Health Organization (WHO) guideline of 10 μg/L. Observations suggested that it was probably because the residents had not followed the operation instructions at the two wells that did not meet the WHO guideline. With the exception of Mn, the average concentrations of other inorganic constituents of health concern (Cr, Ni, Cu, Se, Mo, Cd, Sb, Ba, Hg, Pb, and U) in treated water were below their respective WHO guidelines for drinking water.

### 6.3. Arsenic Removal by Adsorption Based Technologies

Adsorption is one of most common processes used for water treatment. A number of literatures have addressed the removal of arsenic by adsorption via a number of adsorbents [1,2,82], including commercial activated carbon, activated alumina, layered double hydroxide, natural/modified clays and zeolites.

#### 6.3.1. New Development on the As Removal by Iron-doped Activated Carbon (AC)

An iron hydroxide-doped activated carbon was developed [83] in which the iron hydroxide will give the activated carbon an elevated active surface area for arsenic adsorption and also help avoid the blockage of the activated carbon pores. The iron content of the modified samples ranged from 0.73 to 5.27%, with smaller iron hydroxide particles exhibited an enhanced arsenic adsorption capacity. The best arsenic adsorption capacity was reported as 4.56 mg As/g at equilibrium and pH 7.

**Figure 6:**
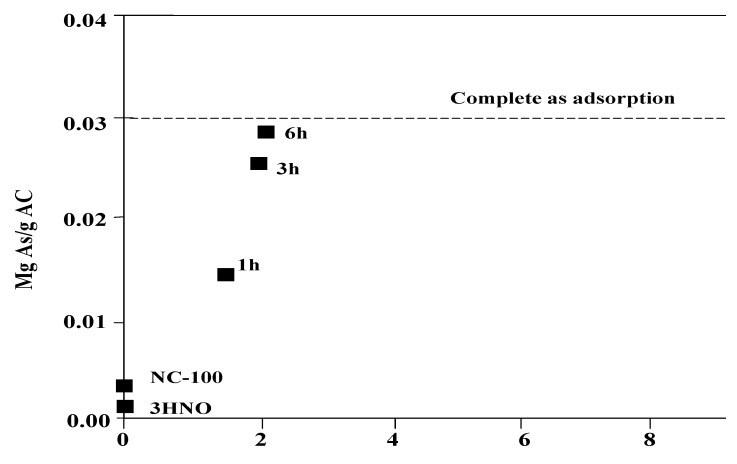
Arsenic uptake of all the investigated materials (raw and iron-doped ACS), as a function of their Fe content [after 84].

In another research project on iron-doped activated carbon [84], iron content increased linearly with iron hydrolysis time. Nanoparticles of iron hydroxide formed at the outer surface of the carbon grains after 6 h hydrolysis time was homogeneous in size and well-dispersed in the carbon matrix. As shown in Figure 6, the 6 h hydrolysis and iron-doped activated carbon (AC) had 2.2% of Fe content on the surface, possessed the highest arsenic adsorption capacity and removed 94% of the arsenic present in a groundwater from the State of Chihuahua (Mexico), whereas the commercial activated carbon (NC-100) allowed the removal of only 14%. Interesting to note that AC prepared using long forced hydrolysis time (24 h) had lower performance in arsenic removal observed (Figure 6); this is probably due to the existence of iron hydroxides agglomerates, which once hydrated could prevent diffusion of arsenate (HAsO_4_^−^) towards the inner surface of the AC grain.

#### 6.3.2. Advances in As Removal by Activated Alumina (AA)

**Figure 7:**
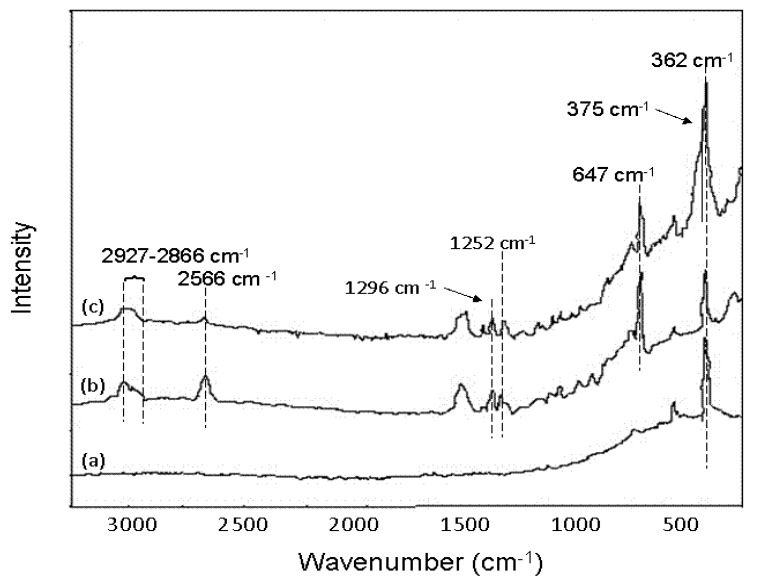
Raman spectra of (**a**) AA, (**b**) hybrid AA before and (**c**) after As(III) adsorption (after [85]).

Activated alumina (AA) has been used widely used for removal of various ions, including arsenic. Recent studies have been made to improve the As removal performance. Various AA hybrid adsorbents bearing thiol groups (-SH) have been prepared by modifying AA with mercaptopropyl-functionalized silica under different experimental conditions [85]. Raman spectra demonstrated the successful functionalization of AA and verified the formation of As-S complexes after As(III) adsorption (Figure 7).

**Figure 8:**
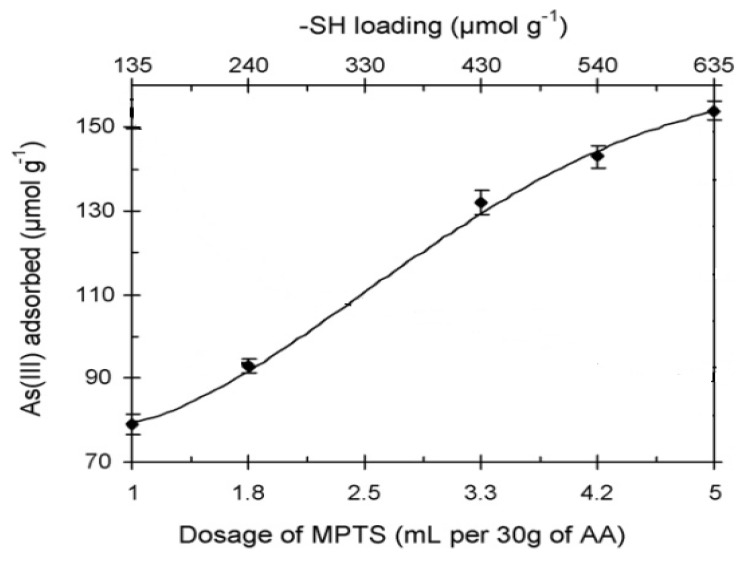
Removal of As(III) as function of -SH loading in the suspensions containing 1.0 g /L adsorbent. Initial As(III) = 20 mg/L, equilibrium pH 7.0 ± 0.1, equilibrium time = 38 h (after [85]).

Compared with AA (spectrum a), the hybrid adsorbent (spectrum b) clearly showed the mercaptan S-H stretching appeared at 2,566 cm^−1^ as a strong band, and the C-H vibration bands of mercaptopropyl groups appeared in the range of 2,927–2,866 cm^−1^. The Raman peaks at 1,296 and 1,252 cm^−1^ assigned to CH2-Si and CH2-S, respectively, were clearly observed. The C-S stretching mode was found at 647 cm^−1^ with a higher intensity. After As(III) adsorption, a strong new shoulder band (overlapped with the band at 362 cm^−1^) appeared at 375 cm^−1^ in the Raman spectrum (c), indicating the formation of the As-S bond. At the same time, the S-H stretching showed a dramatic decrease in intensity. The Raman spectra change provided evidence for the binding of As(III) directly to the thiol groups. Batch experiments were applied to evaluate the As(III) adsorption performance of the hybrid adsorbents. Compared with AA, the hybrid AA exhibited enhanced adsorption abilities for As(III) due to the introduction of thiol groups, and as the thiol loading increased, the uptake of As(III) increased (Figure 8). Based on the results, one hybrid adsorbent referred to as BL(AA)30(MPTS)3.3 has been selected by consideration of not only the adsorption capacity but also its environmentally friendly and cost-effective production.

Inorganic hybrid alumina with copper oxide, namely, copper oxide incorporated mesoporous alumina (COIMA) has been developed for the removal of arsenic from water [86]. The COIMA was prepared by treating mesoporous alumina with copper sulphate solution, followed by calcination at 450 °C in the presence of air. Various adsorption isotherm and kinetic parameters were computed using batch adsorption studies to determine the adsorption capacity for As(III) and As(V) and to understand the mechanism of adsorption. It was observed that incorporation of copper oxide improves the adsorption capacity of unmodified alumina from 0.92 to 2.16 mg/g for As(III) and from 0.84 to 2.02 mg/g for As(V). The results revealed that the adsorption follows Langmuir isotherm and pseudo-second-order kinetic models for both As(III) and As(V). The material is capable of simultaneously removing As(III) and As(V) with removal efficiencies of more than 95% for both As(III) and As(V). Assessment of the water quality before and after treatment with COIMA also confirmed that there is no leaching of copper and other water quality parameters were within permissible limits of Indian drinking water standards.

#### 6.3.3. As Removal by Layered Double Hydroxide (LDH)

Two types of layered double hydroxides (LDHs), hydrotalcite and hydrocalumite with different composition of layers and interlayers were investigated for the removal of arsenite [87] and the study found that arsenite removal was 87.5% and 83.6% respectively, with the nitrate forms of hydrotalcite and hydrocalumite. LDHs synthesized at room temperature showed higher As uptake than those synthesized by hydrothermal method due to small crystal size (and then high surface area) of the former. The uptake process was anion exchange in hydrotalcite-type LDH as confirmed by X-ray diffraction (XRD) and scanning electron microscopy (SEM) but possibly some dissolution-reprecipitation occurred with hydrocalumite-type LDH.

Mg-Fe-based LDH (FeHT) was studied for the sorptive removal of arsenate (As(V)) from aqueous solutions [88]. The FeHT with the chemical formula [Mg(II)_6_Fe(III)_2_(OH)_16_]^2+^[CO_3_yH_2_O]^2^^−^ was prepared by a co-precipitation method. The results have shown that FeHT has a high arsenate removal efficiency, with the ability to reduce the concentration of arsenate in the aqueous solution from an initial value of 330 μg/L to <10 μg/L (*i.e.*, below the limit value specified by WHO).

#### 6.3.4. As Removal by Natural and Modified Zeolites and Clays

Due to their low cost and the capability of ion-exchange and adsorption, zeolites have seen wide application in environmental remediation. Zeolites are alumino-silicate minerals with a three dimensional porous structure based on silica (SiO_4_) and alumina (AlO_4_) tetrahedral configuration. The most abundant natural zeolite is clinoptilolite (empirical formula: (Ca,K_2_,Na_2_,Mg)_4_Al_8_Si_40_O_96_24H_2_O). Depending on the mineralogical content and source of origin, the major chemical composition of natural zeolites includes SiO_2_: 42–75.5%, Al_2_O_3_: 13.3–15%, Fe_2_O_3_: 0.9–11.4%, CaO: 2.5–8.5%, MgO: 0.7–10.3%. One important property of zeolites is their cation exchange capacity (CEC), which mainly depends on the Al content because each Al^3+^ substitution for Si^4+^ in the zeolite framework generates one negative charge on the framework, meaning the greater the Al^3+^ substitutions, the higher the the negative charge of the zeolite.

**Figure 9:**
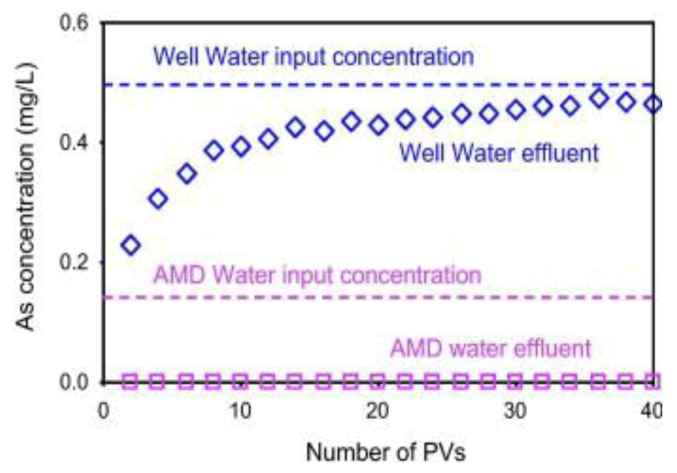
As concentrations from a column test effluent for the acid mine drainage (AMD) wastewater and groundwater (after [68]).

A natural zeolite (clinoptilolite) was modified by iron(III) solutions to enhance its As removal [68]. The As sorption on the Fe-exchanged zeolite (Fe-eZ) could reach up to 100 mg/kg. Columns packed with Fe-eZ were tested for As removal from acid mine drainage and groundwater containing high natural organic matter and high As. Figure 9 shows that for an initial concentration of 147 μg/L in the acid mine effluent, a complete As removal was achieved up to 40 pore volumes (PVs). However, the Fe-eZ was not effective to remove As from groundwater due to its high initial As concentration, with arsenites as dominant species and high amounts of natural organic matter (NOM). In the presence of NOM, arsenite was consistently desorbed or prevented from sorbing to a greater extent than arsenate [89].

In a study [90], the time required to attain equilibrium for arsenic sorption on all types of clinoptilolite was 60 min. However, Fe modified clinoptilolite showed greater As sorption capacity (9.2 μg/g) in comparison with un-modified clinoptilolite, 1.5 μg/g.

**Figure 10:**
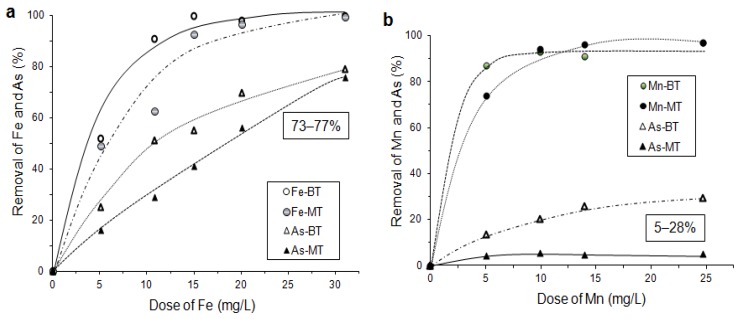
Adsorption efficiency of co-adsorption process; (**a**) Fe/As system, (**b**) Mn/As system; MT-kaolin, BT-bentonite (after [91]).

The use of clays as effective arsenic sorbents has been strongly limited due to their low pHZPC and cation active behaviour in aqueous systems at pH > 3.5. Modifications by Fe/Mn addition can significantly improve their sorption affinity to oxyanions, including arsenites and arsenates. Low grade calcinated kaolin (MT) and bentonite (BT) were used as clay sorbents for the studies [91]. Results demonstrated that arsenic adsorption on raw clays without Fe/Mn modification is very slow and limited. During co-adsorption Fe particles demonstrated a good sorption affinity to the clay surface; up to 77% of As were removed in Fe/As system, in comparison with <30% by the Mn/As system (Figure 10). Arsenic was strongly stabilized in pre-modified sorbents.

#### 6.3.5. As Sorption by Laterite and Limonite with Oxidation

Laboratory and field tests were conducted in rural and sub-urban areas of the Vietnamese capital of Hanoi to assess the suitability of using oxidation processes by activated hypochlorite in water treatment plants and naturally occurring minerals as sorbents in household-based systems to reduce arsenic concentrations [78]. Laterite and limonite, which are naturally and widely occurring minerals in Vietnam, were used as sorbents for arsenic removal in small scale water treatment. The sorption capacities of laterite and limonite for As(V) were estimated to be 1,100 and 900 mg/kg, respectively. Initial results of field tests indicated that arsenic concentrations decreased to levels <0.05 mg/L. The household system based on an adsorption column packed with these minerals seemed to be a suitable technique for small-scale groundwaters remediation in rural and sub-urban areas.

## 7. Discussion

Ground water in 60 out of 64 surveyed districts of Bangladesh is contaminated by As ([As] > 10 µg/L). The most vulnerable regions to As contamination are Satkhira, Munshiganj, Chandpur, Laksmipur, and Noakhali, where 70–90% of the total analyzed groundwater samples have As concentration ≥100 µg/L. Both field-kit and laboratory tests were employed by government and non-government organizations to identify As contaminated water wells. Although field test kits have the limitation of detecting As contaminated groundwater with concentrations between 50 and 200 µg/L [32], both tests provide the same indication of the general geographical distributions of As contamination. Based on the recent literature [19], approximately 22.3% of the 164 million population in Bangladesh was exposed to As contaminated water with [As] > 10 µg/L. One in five deaths in Bangladesh could be attributed to As exposure (>10 µg/L) in drinking water. Such a severe As crisis in Bangladesh has been described as “the largest mass poisoning of a population in history” [9], and thus has led to the development and implementation of feasible treatment technologies to remove As from water.

Although various technologies are available for the removal of arsenic from contaminated water, not all of them fit the local situations in application. Ion exchange and membrane technology could achieve high As removal, but capital investment/operating costs are so high that local communities in the developing countries cannot afford them.

As shown in Table 4, many other technologies have been tested which can be considered as alternatives to the advance technologies. Oxidation/filtration is designed to remove As from iron and manganese containing groundwater. The processes involves the oxidation of the soluble iron and manganese to their insoluble forms and oxidation of As(III) to As(V). Arsenic is removed via co-precipitation with Fe/Mn oxidising species and then filtration. The oxidants could be added hypochlorite or simply via aeration (biological oxidation). This technology has been widely accepted in many developing countries as it achieves high As removal efficiency, requires less investment and needs low operating cost.

Precipitation/co-precipitation is widely used as a conventional technology to treat both drinking water and wastewater. The effectiveness of this technology to treat arsenic is high, together with high treatment efficiency for other pollutants such as heavy metals. However, the sludge production and its handling cost limit its use in household level but it is more cost effective at a large scale where labour costs can be spread over a larger amount of treated water produced.

**Table 4:** Technologies and treatment efficiencies.

Treatment Process As(V)	Removal Efficiency *	As concentration in raw water	Ref.
***Oxidation and Filtration***			
Aeration and filtration	>90%	300 µg As(III)/L	This review
Fe_2_O_3_ filter	>95%	100-400 µg As(III)/L	This review
As(III) oxidation by (OCl^−^) and Fe precipitation	>98%	300 µg As(III)/L	This review
***Co-precipitation***			
Enhanced lime softening	90%		[27]
*Enhanced coagulation/filtration*			
With alum	<90%		[27]
With ferric chloride	95%		[27]
***Adsorption***			
Iron doped activated carbon	>95%	311 µg As/L	This review
Hybrid activated alumina	>95%	2–20 mg As/L	This review
Iron based sorbents	Up to 98%		[27]
Layered double hydroxide (LDH)	Up to 96%	300 µg As(V)/L	This review
Modified zeolites	up to 99%	100–400 µg As/L	This review
Modified clays	Up to 80%	0.15 µM As	This review
Laterite and limonite	Up to 95%	500 µg As/L	This review

* depending on source water composition and operating conditions.

Small capacity systems using adsorption tend to have lower operating and maintenance costs, and require less operator expertise. Adsorption is more effective when arsenic is the only contaminant to be treated. The modification of commercial adsorbents seems to be an approach to improve As removal efficiency.

Iron-doped activated carbon removed 94% of arsenic from ground water in comparison to 14% by commercial activated carbon. Moreover, activated alumina (AA) has been used for more than two decades to decontaminate As(V)-containing waters but is ineffective in As(III) adsorption. Advances in using AA have been made via the preparation of hybrid AA which bears either thiol groups (-SH) or copper oxide and can enhance the As(III) removal efficiency significantly. Such kind of materials are specifically useful in treating As in household levels and in the rural regions of developing countries.

After modification, natural mineral materials (zeolites, clays and laterite and limonite) are also good adsorbents for the removal of As (Table 4). In recent years, layered double hydroxides (LDHs) or hydrotalcite-like compounds have been widely studied [92,93]. LDHs can be represented by a general formula M(II)_1__−x_ M(III)_x _(OH)_2_(An^−^)_x/n_·mH_2_O, in which both divalent [M(II)] (e.g., Mg^2+^, Ca^2+^) and trivalent [M(III)] cations (e.g., Al^3+^, Fe^3+^) give positively charged sheets. The positive charge is balanced by intercalation of anions (An^−^) (e.g., NO_3_^−^, CO_3_^2^^−^) in the hydrated interlayer regions. Based on the structure of LDHs, positive charges are balanced by interlayer anions which can be exchanged for other anions, thus, LDHs possess a good anion exchange property and can be used as good anion-exchangers to remove As (shown in Table 4). Due to their low cost and the high capability of As adsorption, it can be expected that modified natural minerals and LDHs are representative of emerging materials/technologies to be further tested for the full scale application for the arsenic removal in the developing countries.

## 8. Concluding Remarks

Ground water in 60 out of 64 surveyed districts of Bangladesh is contaminated by As ([As] > 10 µg/L). The regions most vulnerable to As contamination are Satkhira, Munshiganj, Chandpur, Laksmipur, and Noakhali, where 70–90% of the total analyzed groundwater samples have As concentrations ≥100 µg/L. Bangladesh is the most affected among the As problem facing countries; up to 77 million people in Bangladesh have been exposed to toxic levels of arsenic from drinking water. Such a severe As crisis has led to the development and implementation of feasible treatment technologies to remove As from groundwater.

Aeration/Fe precipitation/filtration is designed to remove As and the technology has been accepted in Bangladesh as it achieves high As removal efficiency, requires less investment and has low operating costs. Small capacity adsorption systems tend to have lower operating and maintenance costs, and require less operator expertise. The modification of commercial adsorbents (e.g., activated carbon and activated alumina) and modification of natural mineral materials (e.g., zeolites) seem to be an approach to lower the operating cost and improve As removal efficiency. While a number of technological developments in the arsenic removal field have taken place, one should bear in mind that the technologies described in this review each have their own advantages and disadvantages. In the implementation of these technologies, we must consider variations in sources and quality characteristics of As polluted water and differences in socio-economic and literacy conditions of people, and then aim at improving the effectiveness in arsenic removal, reducing the capital and operation costs of the systems, making the technology user friendly, overcoming maintenance problems and resolving sludge management issues.
